# Supplementary material for: The Construction and Exploration of a Comprehensive MicroRNA Centered Regulatory Network in Foxtail Millet (Setaria italica L.)
Source: Front Plant Sci. 2022 May 6;13:848474. doi: 10.3389/fpls.2022.848474 (PMC9121102; doi:10.3389/fpls.2022.848474)
Supplement: Supplementary file 3 [file Data_Sheet_3.zip › Sit-miRN3000_Seita.1G029100.1_656_TPlot.pdf]

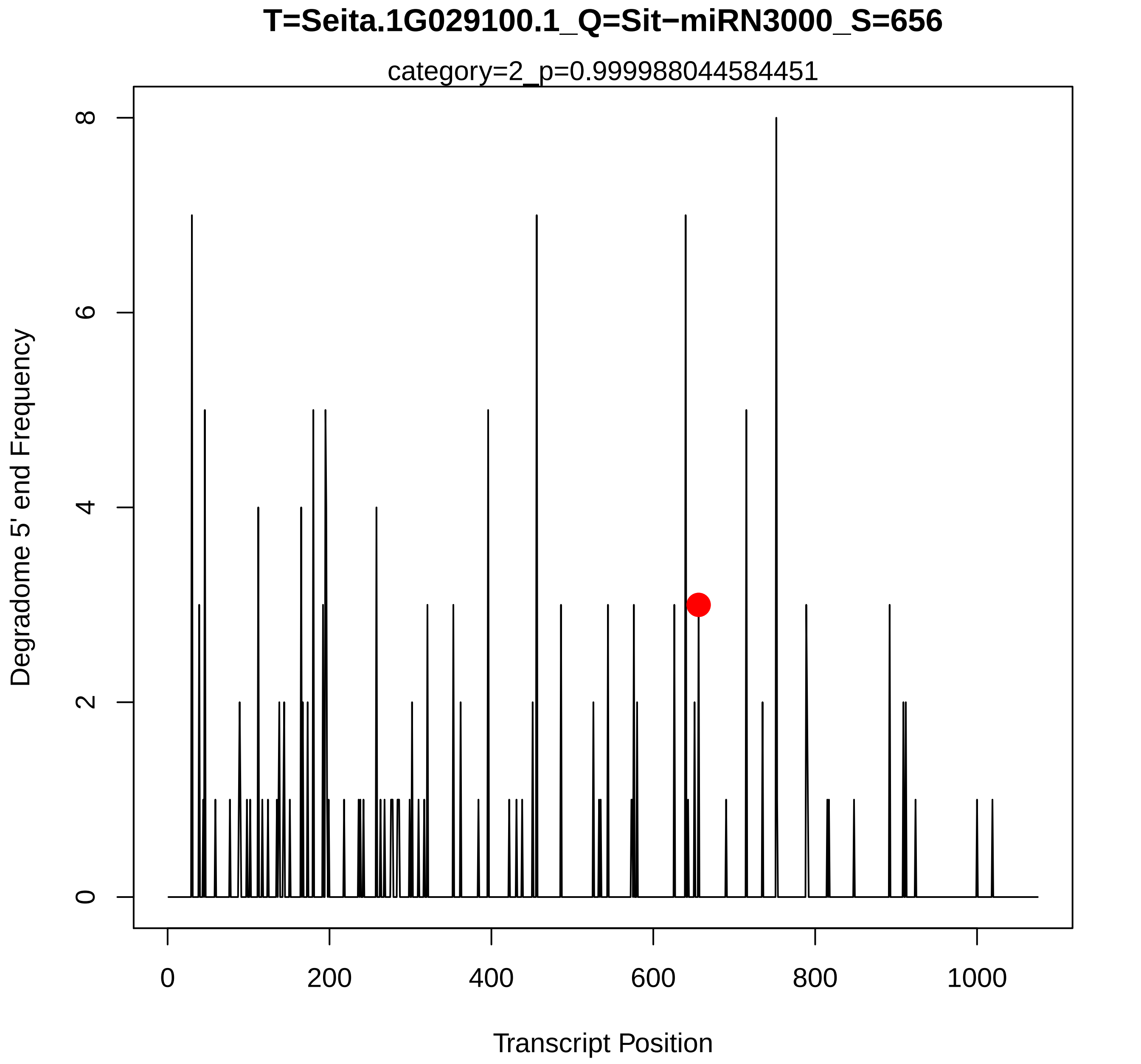

T=Seita.1G029100.1_Q=Sit−miRN3000_S=656
category=2_p=0.999988044584451
Degradome 5' end Frequency
Transcript Position
0
2
4
6
8
0
200
400
600
800
1000
